# Functional *in vitro* assessment of modified antibodies: Impact of label on protein properties

**DOI:** 10.1371/journal.pone.0257342

**Published:** 2021-09-16

**Authors:** Martin R. Edelmann, Simon Hauri

**Affiliations:** 1 Department of Pharmacy and Pharmacology, University of Bath, Bath, United Kingdom; 2 Roche Pharma Research and Early Development, Roche Innovation Center Basel, F. Hoffmann-La Roche AG, Basel, Switzerland; Consiglio Nazionale delle Ricerche, ITALY

## Abstract

Labelling of therapeutic antibodies with radionuclides or fluorophores is routinely used to study their pharmacokinetic properties. A critical assumption in utilizing labelled therapeutic antibodies is that the label has no unfavourable effects on antibody charge, hydrophobicity, or receptor affinity. Ideally, the labelled protein should not have any significant deviations from the physiological properties of the original molecule. This article describes an established quality *in vitro* assessment workflow for labelled antibodies that ensures better prediction of changes in antibody pharmacokinetic (PK) properties after modifications. This analysis package considers degradation and aggregation analysis by size-exclusion chromatography, changes in neonatal-Fc-receptor (FcRn) affinity, and heparin interaction. FcRn binding is important for antibody recycling and half-life extension, whereas heparin affinity provides estimates on the rate of endocytosis through unspecific cell surface binding. Additionally, mass spectrometric analysis to determine the degree of labelling (DoL) completes the package and the combined analysis data allow to predict the label contribution to the PK properties of the modified antibody. This analytical strategy for labelling 11 IgGs has been investigated using 2 different IgG_1_ constructs and applying 7 different types of labels. Each labelling resulted in a change in the physicochemical properties of the protein. Not only can the DoL of modified IgGs lead to a change in protein properties, but the type of label also can. Furthermore, it was demonstrated that the labelling process can also influence the behaviour of labelled mAbs. An identical label on different constructs of IgG_1_ can cause different affinities for FcRn and heparin. Considering the assessment data, only 6 of the 11 modified antibodies from this study can be recommended for subsequent experiments. In conclusion, a suitability assessment of labelled antibodies prior to any pharmacokinetic studies is essential to reduce cost, allocate resources and reduce the number of animal experiments during pre-clinical drug development.

## Introduction

In drug discovery and development of therapeutic antibodies, the implementation of protein labelling techniques is an extremely valuable tool for *in vitro* and *in vivo* testing to gain a better understanding the fate of therapeutic proteins in the body [1]. Radioactive or fluorescent labels attached to monoclonal antibodies (mAbs) or antibody fragments are routinely used in preclinical development, e.g. in biotransformation [2], biodistribution [3], or binding [4] studies. The labels allow tracking, monitoring, and imaging mAbs within complex biological matrices to evaluate their stability and disposition. Several types of labels are available, largely differentiated in fluorescent dyes for light and electron microscopy, and radioactive isotopes for imaging applications. Both can also be analysed by high-performance liquid chromatography (HPLC) combined with the appropriate detectors. It should be considered that the introduction of labels poses a risk of changes in the physicochemical properties of the labelled protein. Not only can the degree of label (DoL) change the properties of modified IgGs [5], but the type of label may as well. For example, fluorescent labels often contain sulfonic acids, which upon conjugation add negative charges to the surface of the protein. Even more drastic, the labelling process could rely on harsh reaction conditions, such as oxidation or reduction, and damage the structure of the antibody entirely.

A quality control workflow was established which, in addition to the classic purity determination by size-exclusion chromatography (SEC), assesses changes in cell-specific receptor affinities and surface charges of the labelled protein. The involved analytical methods are SEC, neonatal Fc receptor (FcRn) and heparin affinity chromatography, as well as intact mass spectrometry (MS). This article describes the quality control workflow for labelled antibodies and compares the analytical results with their unlabelled counter-parts. It is not covering protein modifications for radioimmuno conjugates [6] or antibody drug conjugates [7] in which a radiolabel or toxic payload is desired. The main question addressed in this study is whether the label has an impact on the pharmacokinetic (PK) properties and how they can be extrapolated back to the parent antibody.

### Labelling of amino acid residues

**Fig 1:**
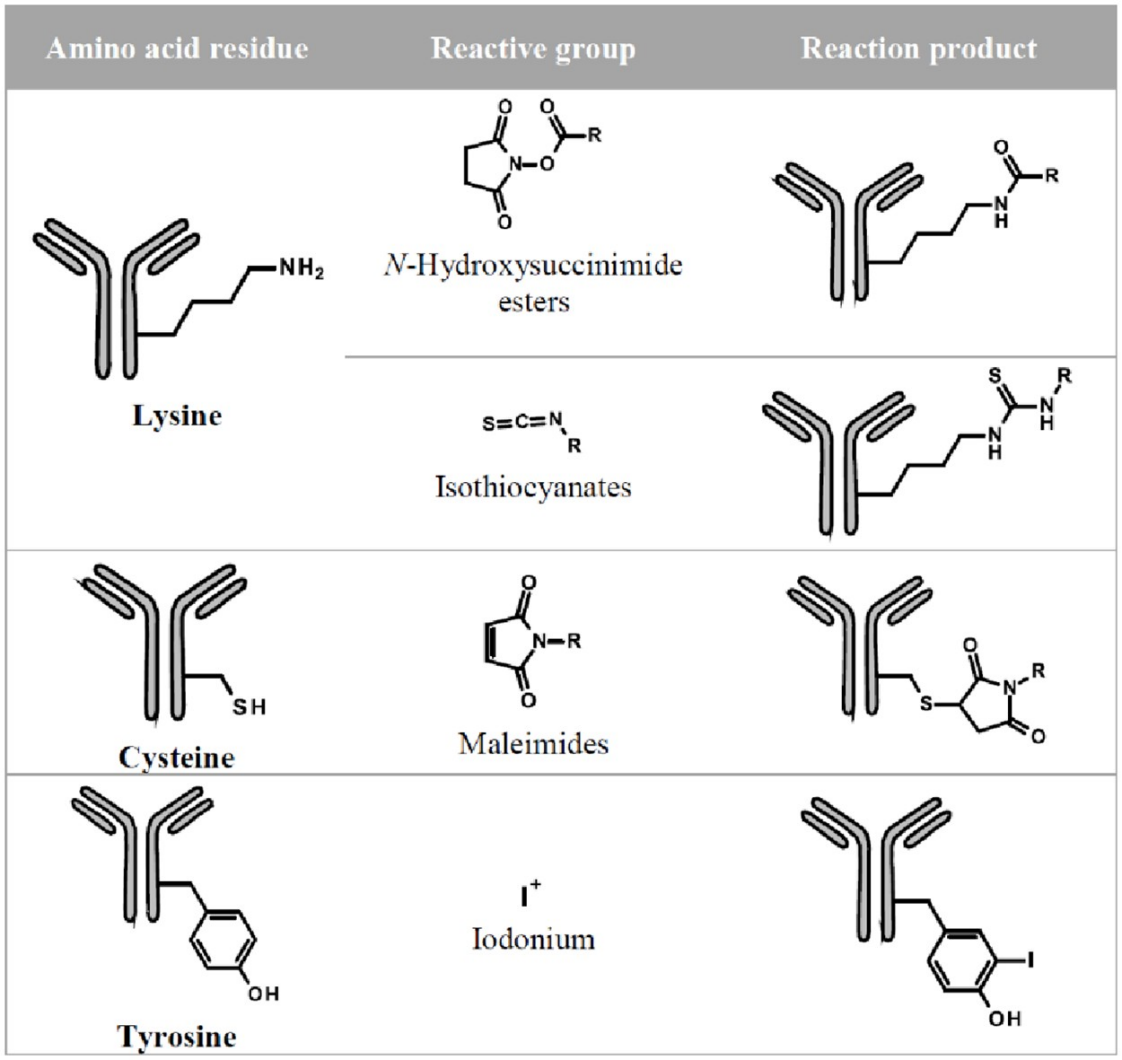
Overview of labelling techniques used in this study. Amino acid residues schematically represent the majority of the amino acids on which labelling or modification takes place. Reactive group describes the part of the label that conjugates with the corresponding amino acid side chain. The reaction product shows the formed chemical structural formula after the covalent linkage of the label to the protein.

A successful protein labelling technique depends on two chemically compatible requirements: A reactive group on a derivatisation reagent and surface accessible functional groups from amino acids in the antibody. This study included two types of protein labelling: 1) the direct introduction of radioactive atoms, e.g. ^125^I to functional groups without the use of chemical spacers, and 2) by conjugation on functional groups in the amino acid sequence of proteins using reactive tags. An overview of the labelling techniques used for this study is given in Fig 1.

### Conjugation of lysine residues

There are around 80 lysine residues on average across a humanized monoclonal IgG_1_ protein. Peptide-mapping experiments could identify almost 40 lysine residues for a potential conjugation [8]. *N*-Hydroxysuccinimide esters (NHS) and isothiocyanates (SCN) are the most common reactive groups for a protein modification on ε-amino group of lysine residues or the *N*-terminal α-amine group. NHS ester-containing reagents react with amines in a pH range of 7–8 to form a stable amide bond. *N*-Succinimidyl propionate (NSP) was selected for this study since the corresponding tritium variant of NSP [9] is suitable for a fast and efficient technique for incorporating the radio isotope tritium into a protein. In contrast to NHS reagents, the electrophilic carbon of the isothiocyanate-group reacts almost selectively with amines to form a stable thiourea. A consequence of amine conjugation is the loss of the positive charge from lysine residues, which may have an impact on (at least) unspecific binding to the cell surface.

### Conjugation of cysteine residues

An alternative to conjugation of lysine residues is the introduction of maleimide-based labels on cysteines [10]. Cysteine residues form intramolecular disulfide bridges, which stabilize the protein tertiary structures. Disulfides do not react with maleimides. Therefore, it is necessary to reduce disulfides prior to the conjugation. Reducing agents such tris(2-carboxyethyl) phosphine (TCEP) can break disulfide bonds, which then can be used for a maleimide-containing labelling modification. This labelling process, however, can lead to damage of the protein.

### Iodination of tyrosine residues

Radioiodination of proteins has a long tradition [11, 12]. Wilbur [13] reported a detailed overview about radiohalogenation of proteins including various methods and reagents for conjugate labelling. An *in situ* generated mixture of halogen iodo-chloride species performs an electrophilic substitution of hydrogen under oxidative conditions on tyrosine or histidine residues. The sites of radioiodination vary with the choice of oxidizing agent and the pH of the labelling reaction. In general, formation of iodinated tyrosyl residues predominates near pH 7 and yields primarily monoiodotyrosine [14]. Non-radioactive sodium iodide was used for the test labelling experiments in order to avoid working with specialist equipment and laboratory facilities with regards to radiation safety.

## Materials and methods

### Chemicals and reagents

Antibodies for this study were produced in house. Alexa Fluor 488 (AF488)-Maleimide and Alexa Fluor 488-NHS were purchased from Invitrogen (Carlsbad, CA, USA), thulium (III) 2-(4-isothiocyanatobenzyl)-1,4,7,10-tetraazacyclododecane-1,4,7,10-tetraacetic acid (Tm-*p*-SCN-Bn-DOTA) from Macrocyclics (Plano, TX, USA). *N*-Succinimidyl propionate (NSP) was purchased from Wako-Chemicals (Richmond, VA, USA), DOTA-NHS from Synchem (Felsberg / Altenburg, Germany), and the water-soluble Bolton-Hunter reagent sulfosuccinimidyl-3-(4-hydroxyphenyl)-propionate sodium salt (Sulfo-SHPP) from Apollo Scientific (Cheshire, UK). Sodium iodine (Sigma-Aldrich Chemie, Munich, Germany) and Pierce pre-coated IODO-GEN iodination tubes (Thermo Fisher Scientific, Waltham, MA, USA) as an oxidizing agent were used for iodination experiments. All reagents were used without further purification.

### Analytical equipment

Protein concentrations were determined by Eppendorf (Hamburg, Germany) BioSpectrometer^®^ basic in combination with an Eppendorf μCuvette^®^ G1.0 with 1 mm path length at 280 nm wavelength and the corresponding calculated molar extinction coefficient. For the fluorescence-labelled mAbs, the absorbance at 494 nm was measured to calculate the degree of labelling with AF488 dye. Chromatographic analysis for size-exclusion, FcRn affinity, and heparin affinity was performed on an Agilent 1200 series HPLC system (Santa Clara, CA, USA). Agilent ChemStation software was used for data evaluation.

### Labelling procedure

**Fig 2:**
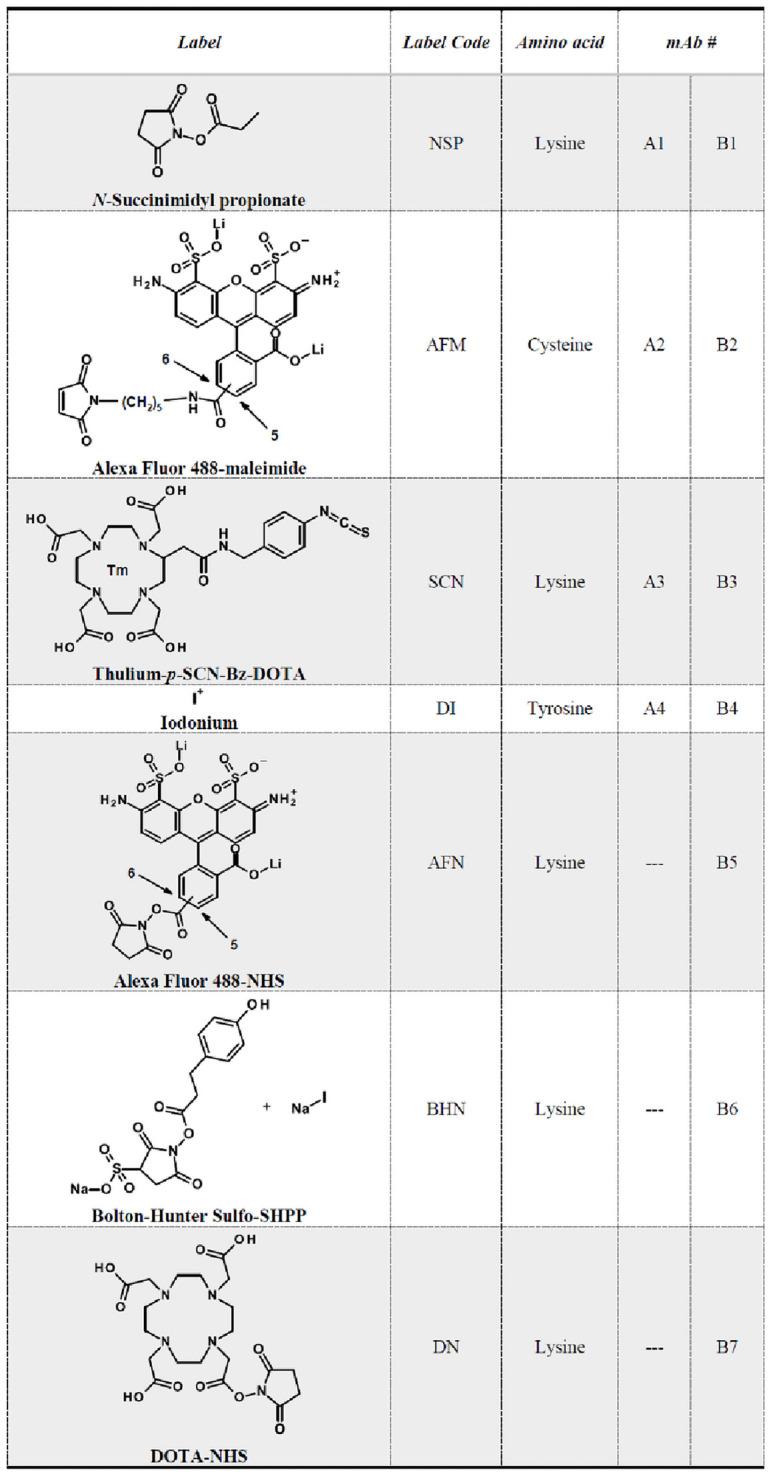
Labels used in this study. Chemical structures of labels used in this study. Label Code describes the abbreviations of each label mentioned in this study. Column “Amino acid” shows the corresponding amino acid involved in the labelling process. Alphanumeric links to the modified mAbs are given in the two right-hand columns: mAbA1-4: wildtype mAb; mAbB1-7: research concept mAb.

In this study, 2 different subclass 1 immunoglobulin G (S1 Sequences) were modified with various labels. mAb_A_ is a human wild-type antibody, the second protein mAb_B_ is a concept antibody with reduced affinity to Fc-gamma receptor Fc-γR (LALA-PG mutation) [15]. An overview of the antibodies with different type of labels and the corresponding chemical structures is given in Fig 2. The aim was to obtain 1 to 3 labels per protein with all labelling techniques in order to achieve the highest comparability.

*N*-Hydroxysuccinimide (NHS) modifications were carried out in analogy to previously published procedures [16]. Briefly, an IgG solution in PBS (protein concentration: 1 to 5 mg/mL; pH 7.5 to 8.5) was added to NHS-based labels (3 molar equivalents, dissolved in DMSO), and shaken orbitally for 30 minutes.

mAbs, conjugated with Alexa Fluor 488-maleimide, were labelled strictly according to Invitrogen’s kit-manuals: *Thiol-Reactive Probes*. In summary, reduction of thiol-residues with tris(2-carboxyethyl) phosphine (TCEP, 10 equivalents) in PBS pH 7.5 and a protein concentration of 2 mg/mL. Maleimide (11 equivalents in DMSO) was added to the protein solution and incubated for 2 hours.

Conjugations with Tm-*p*-SCN-Bn-DOTA (20 equivalents in DMSO) was performed in HEPES (4-(2-hydroxyethyl)-1-piperazineethansulfonic acid (Gibco, Paisley, UK), at pH 7.5 by a protein concentration of 20 to 30 mg/mL by mild shaking for 3 hours.

Direct iodination on tyrosine residues was carried out according to Thermo Fisher Scientific manual 0016379; Example Protocol II: *Direct Method for Iodination*. Indirect iodination, using water soluble Bolton Hunter-NHS reagent, was performed in analogy to Thermo Fisher Scientific’s Example Protocol III: *Iodination of Crosslinkers*. Pierce^™^ Pre-Coated Iodination Tubes (Iodo-Gen^®^) were used for both iodination techniques, direct and indirect.

All labelled antibodies were buffer exchanged into 20 mM histidine, 140 mM sodium chloride, pH 6 formulation buffer using a PD MidiTrap G-25 desalting cartridge (GE Healthcare, Chicago, IL, USA). The DMSO volume of the label solutions never exceeded 5% of the total volume. Solutions for fluorescence labelling were protected from light as much as possible by wrapping all containers in aluminum foil.

### Chromatography

Whenever possible, a common baseline was drawn and the peaks were split at minima or plateaus. A plateau is reached at a position where the chromatogram is parallel to the baseline. The highest peak retention time was used for the evaluation. Chromatograms of all mAbs used in this study can be found in S1–S3 Figs.

### Size-exclusion chromatography

Samples were analysed using a TSKgel G3000 SW_XL_ column (Tosoh Bioscience, Tokyo, Japan), 7.8 x 300 mm, 5 μm with 0.2 M potassium phosphate, 0.25 M potassium chloride, pH 7.0 as the mobile phase at a flow rate of 0.5 mL/min. Absorbance at 280 nm and in addition 494 nm for the fluorescence labelled mAbs were used for detection and quantification. The injection volume was 10 μL and the protein concentration 1 mg/mL. The target concentration of 1 mg/mL was set by adding the eluent to the protein stock solution.

### FcRn affinity chromatography

Analytical FcRn affinity chromatography [17] was carried out with an FcRn affinity column (Roche Custom Biotech, Mannheim, Germany), column volume of 0.5 mL containing 1.5 mg FcRn protein. For detection and quantification, an absorbance at 280 nm and in addition 494 nm for fluorescence labelled mAbs were used. The 45-min continuous gradient was applied with a flow rate of 0.5 mL/min (Table 1). The injection volume was 30 μL and the protein concentration 1 mg/mL. The target concentration of 1 mg/mL was set by adding eluent A to the protein stock solution.

**Table 1:** Chromatographic method used for FcRn affinity chromatography.

*Time [min]*	*Eluent A [%]*	*Eluent B [%]*
0	80	20
5	80	20
40	0	100
45	0	100
46	80	20
51	80	20

Eluent A: 20 mM 2-(*N*-morpholino)ethanesulfonic acid (MES), 140 mM NaCl buffer, pH 5.5; Eluent B: 20 mM tris(hydroxymethyl)aminomethane (Tris), 140 mM NaCl buffer, pH 8.8.

### Heparin affinity chromatography

A commercially available heparin column (TSK-Gel Heparin-5PW, 5 x 50 mm, Tosoh Bioscience, Tokyo, Japan) was equilibrated with eluent A, followed by a 23 min continuous gradient to 100% eluent B was applied with a flow rate of 0.8 mL/min (Table 2). The injection volume was 30 μL and the protein concentration 1 mg/mL. The target concentration of 1 mg/mL was set by adding eluent A to the protein stock solution.

**Table 2:** Chromatographic method used for heparin affinity chromatography.

*Time [min]*	*Eluent A [%]*	*Eluent B [%]*
0	100	0
2	100	0
18.5	45	55
19	0	100
23	0	100
24	100	0
32	100	0

Eluent A: 50 mM Tris, pH 7.4; Eluent B: 50 mM Tris, 1 M NaCl, pH 7.4.

### Mass spectrometry

For mass spectrometry analysis, a Waters (Baden, Switzerland) nanoAcquity pump equipped with Waters LCT Premier XE and a Acquity UPLC Protein C4 column, 300 Å, 1.7 μm, 1 x 50 mm was used in ESI positive mode at a flow rate of 70 μL/min. Mobile phases used were (A) water/acetonitrile 9:1 with 0.1% trifluoroacetic acid and (B) acetonitrile/water 9:1 with 0.1% trifluoroacetic acid, from 10% (B) to 65% (B) within 10 minutes. Proteins have been analysed (if not otherwise mentioned) reduced and deglycosylated by the use of Rapid PNGase F (New England Biolabs, Ipswich, MA, USA) according to the following representative procedure: 5.7 μL miliQ water was added to 10.3 μL (12 μg) protein solution. 4 μL Rapid PNGase F buffer, containing dithiothreitol (DTT) for mild reduction of disulfide bonds (5x) and 1 μL Rapid PNGase F [18] for deglycosylation was added. The mixture was incubated at 50°C for 15 minutes.

## Results and discussion

This section explains the quality assessment package that compares antibodies, which have been modified with different type of labels, to the original, unlabelled parent proteins. The degree of labelling was calculated using mass spectrometry and supplemented by light spectroscopy in case of fluorescence-labelled variants. Size-exclusion chromatography provides a quantitative assessment of the degradation and aggregation of intact proteins. The affinity for neonatal Fc receptor and heparin were carried out using commercially available chromatography columns and the resulting retention times of labelled versus unlabelled protein were compared. These results allowed a correlation with both kinds of affinity chromatography and a prediction of the influence of the label on a change in PK properties of the protein.

### Mass spectrometry analysis

**Fig 3:**
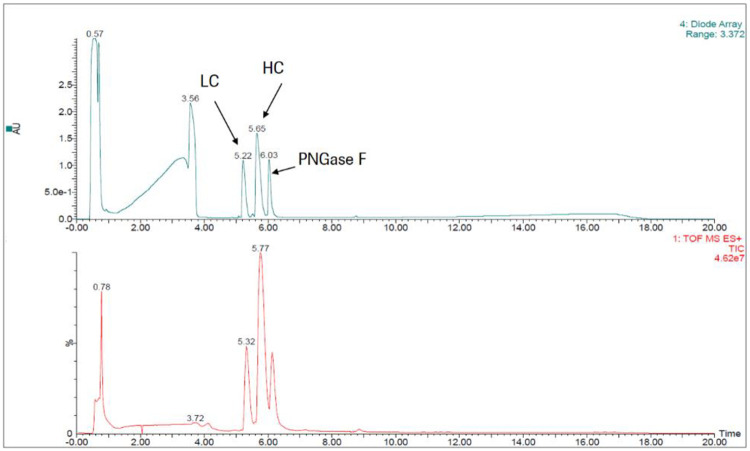
Representative example of a chromatographic separation for subsequent mass spectrometric analysis. LC: light chain; HC: heavy chain; PNGase F: peptide *N*-glycosidase F.

**Fig 4:**
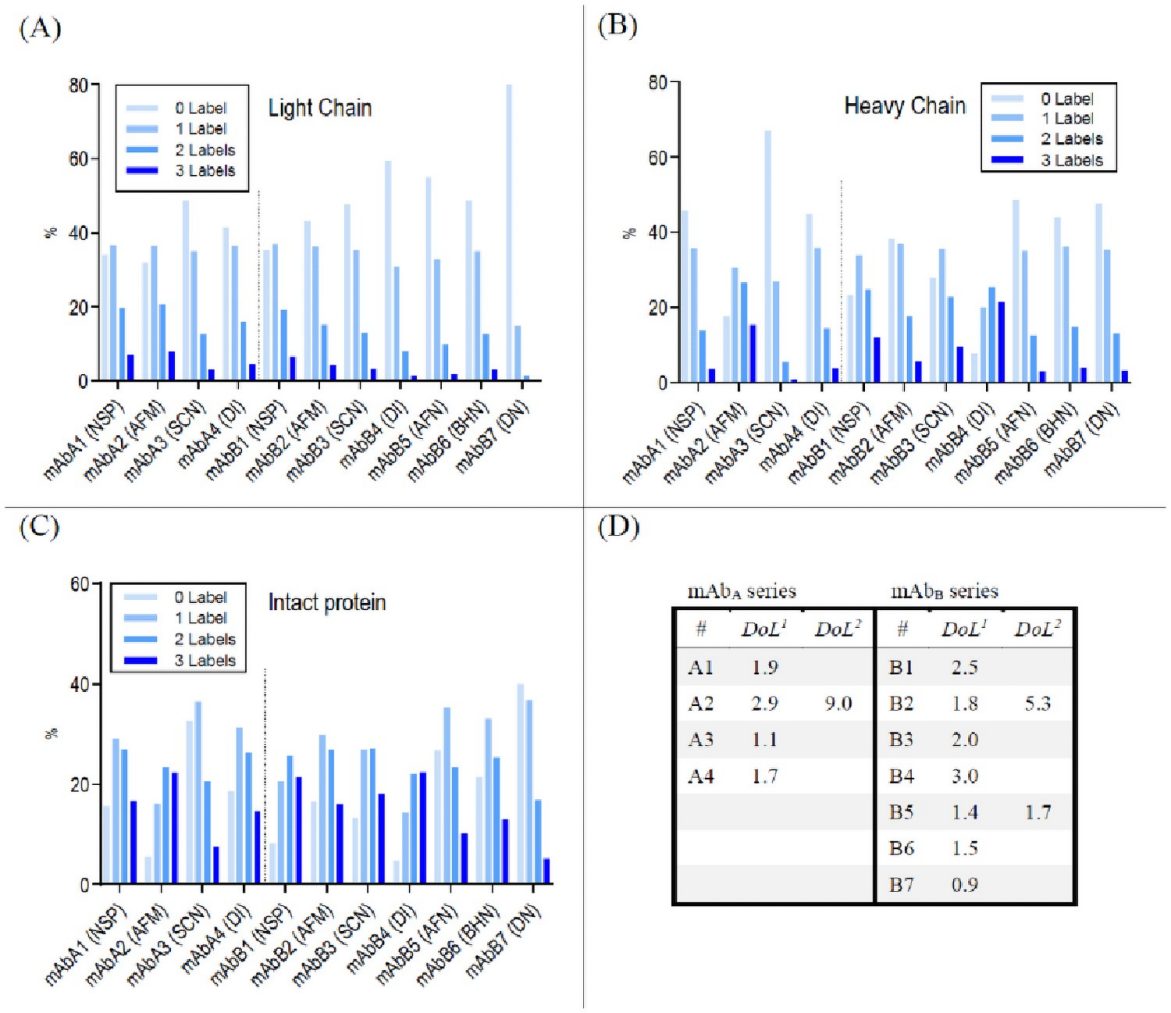
Labelling distribution of mAbA1-4 and mAbB1-7. (A): light chain; (B): heavy chain; (C) intact protein, according to Poisson distribution; (D): calculated degree of labelling (DoL). DoL^1^: based on labelling distribution determined by mass spectrometry analysis; DoL^2^: based on spectroscopic calculation for fluorescence labelled mAbs (Supplementary Information: S2 Formula). Label Code: NSP: *N*-succinimidyl propionate; AFM: Alexa Fluor 488-maleimide; SCN: Tm-*p*-SCN-Bz-DOTA; DI: direct iodination; AFN: Alexa Fluor 488-NHS; BHN: Bolton-Hunter-NHS; DN: DOTA-NHS.

The incorporation of labels at the proteins were identified by mass spectrometry. All labelled IgGs were analysed in reduced and deglycosylated form. Using this method, the heavy chain and the light chain are separated from each other by HPLC measured by intact mass spectrometry (Fig 3). By assuming a Poisson distribution, the percentage of incorporated labels could be determined on both the light and heavy chain. The degree of labelling (DoL) describes how many labels in average are bound to a protein. For example, a DoL of 1 refers to a label:protein molar ratio of 1:1. Considering the Poisson distribution (S1 Formula), the actual number of labels conjugated to an antibody at a DoL of 1 reflect 37% of the antibody molecules are unlabelled, 37% will contain one label, 18% will contain two labels, 6% will contain three labels, and 2% four or more labels. The theoretical calculations from the Poisson distribution are also reflected in the label pattern of the conjugated intact antibody samples from the total ion chromatogram (Fig 4C). Considering the labelling distribution on the light chain and heavy chain of each conjugated antibody (S1 Table), the degree of labelling of the intact protein was calculated and listed in Fig 4D.

DoL determination of mAbB6, labelled with Bolton-Hunter-NHS (BHN) reagent, was more complex, as two different labelling steps were required: 1) the labelling of BHN with iodine and 2) the conjugation to lysine residues using NHS-technology. The iodination of BHN did not result in a homogenous product, but rather a mixture of uniodinated, singly, and doubly iodinated BHN. Mass spectrometric analyses consequently showed a mixture of Bolton-Hunter-conjugate with no iodine, single and double iodine. Regardless the number of iodine on BHN, it was considered as one label when calculating the DoL for mAbB6.

In addition to mass spectrometry analysis, the degree of labelling of the fluorescence-labelled samples was calculated by light spectroscopy using S2 and S3 Formula. The results of mAbB5, Alexa Fluor 488-NHS conjugation, from MS and spectroscopy analysis are comparable. An analytical deviation was encountered in the determination of DoL of mAbA2 and mAbB2, which were conjugated using the maleimide-cysteine technique. The result from the spectroscopy shows a 3-fold higher DoL than calculated by mass spectrometry (S2 Table). An additional MS analysis of proteins without prior reduction during sample preparation provides an explanation: almost no intact protein was found in these analysis. Most of the mass signals showed protein fragments derived from a labelled light chain as well as labelled IgG without a light chain or without both light chains. The protein damage was most likely caused by the labelling process. After the partial reduction of inter-chain disulfide bonds and the subsequent addition of maleimide-dyes to cysteine residues, the corresponding thiols are blocked to form a disulfide bond again, however the quaternary structure is kept intact through inter-molecular forces.

Another major observation was that the incorporation rate of bulky labels on lysine residues is different for the light and heavy chains of the antibody. Alexa Fluor 488 (mAbB5) and DOTA (mAbB7), conjugated by NHS chemistry, showed a lower label incorporation in light chains compared to isothiocyanate based conjugated Tm-DOTA in mAbA3 and mAbB3. This is in particularly noteworthy that NHS and SCN functionalized labels react to lysine residues. *N*-Succinimidyl propionate as a small label in contrast, a higher incorporation at the light chain was observed (Fig 4A and 4B).

### Size-exclusion chromatography (SEC)

SEC is an established application for the routine monitoring of therapeutic proteins with respect to purity and the identification of low as well as high molecular weight impurities [19]. The retention time is a function of the HPLC flow rate, column pore size, and the hydrodynamic radius of the protein, and its potential fragments and aggregates. After conjugating a label to an antibody, the molecular weight does not change dramatically. A change in the hydrodynamic radius, in contrast, can induce a shift in migration time even with a negligible increase in molecular weight [20]. This phenomenon is observed in particular by introducing multiple charged labels such as chelators or fluorescence dyes. The difference in the retention times of the modified antibodies from this study are, however, too low to give a meaningful trend (Table 3).

**Table 3:** Retention times in size-exclusion chromatography.

*mAb #*	*Label Code*	*Retention time [min]*	*mAb #*	*Label Code*	*Retention time [min]*
mAb_A_	---	16.1	mAb_B_	---	15.5
A1	NSP	16.1	B1	NSP	15.5
A2	AFM	16.1 ^(a)^	B2	AFM	15.5 ^(b)^
A3	SCN	16.0	B3	SCN	15.4
A4	DI	16.2	B4	DI	15.5
			B5	AFN	15.5
			B6	BHN	15.6
			B7	DN	15.5

^(a)^: Peak area of 67%, additional peak at 22.6 min (33%).

^(b)^: Peak area of 49%, additional peak at 22.4 min (51%).

Label Code: NSP: *N*-succinimidyl propionate; AFM: Alexa Fluor 488-maleimide; SCN: Tm-*p*-SCN-Bz-DOTA; DI: direct iodination; AFN: Alexa Fluor 488-NHS; BHN: Bolton-Hunter-NHS; DN: DOTA-NHS.

**Fig 5:**
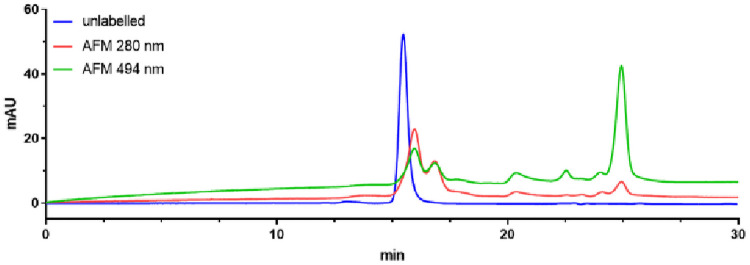
SEC of mAbB2 (Alexa Fluor 488-maleimide), denatured with 0.1% formic acid. Blue line: unlabelled mAb_B_ showed a single peak. Red line: absorbance of modified mAb6 at 280 nm. Green line (494 nm) showed several peaks that indicate (labelled) protein fragments.

Antibodies that have been treated with maleimide-based labels show an obvious degradation and confirm the finding from mass spectrometry of a fragmented antibody. The corresponding SEC shows a peak at the expected migration time of the unlabelled antibody at 16.1 min (mAbA2) and 15.5 min (mAbB2). An additional protein fragment with absorbance at 494 nm at around 22.4 min appeared. Although the molecular weight decreases due the cleavage of light chains, the retention times at around 16 min remains almost the same. The relatively unaltered retention time of the antibody fragments to the unlabelled proteins can be explained by an increase of the hydrodynamic sizes of human IgGs, which was observed after partial reduction of inter-chain disulfide bonds [21]. In an additional SEC experiment, maleimide labelled mAbB2 was treated by the addition of 0.1% formic acid (protein solution to formic acid 1:1 v/v) prior to injection. The main peak (mAbB2) at 15.5 min has disappeared and several peaks eluted at 16.0 min, 16.8 min, 20.4 min, 24.1 min, and 24.9 min (Fig 5). So, possibly, under non-denaturing conditions of the SEC, the reduced antibody was stabilized through a non-covalent interaction (ionic, hydrogen bonds, Van der Waals, or hydrophobic) and remained as intact protein at 15.5 min. However, after denaturation at low pH, the molecule divided into individual chains. This indicates that the disulfide bridges could not be reestablished after the labelling. Free thiol groups may have an unexpected impact on the PK properties of the therapeutic protein and should be avoided if possible.

### FcRn affinity chromatography

The neonatal Fc receptor (FcRn) is expressed by many cells types all over the body and structurally related to class I major histocompatibility complex (MHC I) [22]. The receptor is able to bind IgGs in a strictly pH-dependent manner with high affinity at pH 6 in the acidified endosome and low affinity at physiological pH of 7.4 in plasma [23]. The IgG:FcRn complexes are recycled back to the surface of the cell, whereas unbound proteins undergo lysosomal degradation.

For the setup of the antibody quality control package, we used an affinity chromatography based method [24], which can sensitively detect differences in the pH-dependent interaction between IgGs and FcRn. The chromatography column contains biotinylated heterodimeric FcRn, immobilized onto sepharose streptavidin beads. The protein elution, driven by a pH gradient, resolves protein species according to their pH-dependent affinity to FcRn in a process that mimics the events that take place when recycling endosomes emerge at the cell surface. The result is an antibody-specific retention time. An early elution from the column predicts low affinity to the receptor at endosomal pH and thus a potentially short serum half-life. In contrast, late elution from the column represents a high affinity and a potential long serum half-life, if the pH level is still below 7.4 [25]. After comparing the retention times (Table 4), the results of two labelling techniques are particularly noticeable. First, for IgGs that were labelled by direct iodination, 2 additional peaks appear with a lower retention time. The higher the DoL (mAbA4: 1.7 and mAbB4: 3.0), the more the peak ratio shifts to the left in the chromatogram, which indicates a lower affinity for the neonatal Fc receptor. Apparently, during the labelling process with IODO-GEN, not only was the iodine ion oxidized to form the reactive iodo-chloride species, but the antibody was oxidized within the FcRn binding interface as well. Two surface exposed methionine residues located at position 252 in the C_H_2 domain and position 428 in the C_H_3 domain (EU numbering [26]) interact with the IgG binding region on cellular neonatal Fc receptor. Oxidation of Met252 and Met428 was shown to impair affinity to FcRn and consequently change PK properties of IgGs [27, 28].

**Table 4:** Retention times in FcRn and heparin affinity chromatography.

*mAb #*	*Label Code*	*Retention time FcRn [min]*	*Retention time Heparin [min]*
mAb_A_	---	21.6	5.7
A1	NSP	21.4	5.2
A2	AFM	21.4 ^(a)^	5.4
A3	SCN	21.5 ^(b)^	5.5
A4	DI	20.5 ^(c)^	5.6
mAb_B_	---	20.1	5.1
B1	NSP	20.2	5.1
B2	AFM	20.4 ^(d)^	2.2
B3	SCN	22.4 ^(e)^	5.6
B4	DI	16.5	5.3
B5	AFN	20.1	5.1
B6	BHN	20.3	5.1
B7	DN	20.2	5.1

^(a)^: Peak area of 52%, additional peaks at 2.3 (10%), 2.1 min (9%), and 1.2 min (27%).

^(b)^: Peak area of 76%, additional peak at 20.3 min (24%).

^(c)^: Peak area of 41%, additional peaks at 21.6 min (47%), and 18.8 min (12%).

^(d)^: Peak area of 45%, additional peaks at 3.1 min (18%), 2.1 min (7%), and 1.3 min (35%).

^(e)^: Peak area of 74%, additional peak at 21.0 min (26%).

Label Code: NSP: *N*-succinimidyl propionate; AFM: Alexa Fluor 488-maleimide; SCN: Tm-*p*-SCN-Bz-DOTA; DI: direct iodination; AFN: Alexa Fluor 488-NHS; BHN: Bolton-Hunter-NHS; DN: DOTA-NHS.

**Fig 6:**
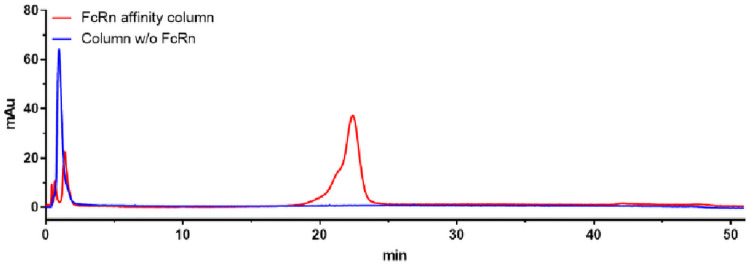
Negative control of mAbB3. Negative control of mAbB3 (Tm-*p*-SCN-Bz-DOTA) using sepharose column without FcRn (blue). The red line shows mAbB3 measured with FcRn affinity chromatography.

A second anomaly was found in chromatograms derived from antibodies conjugated with isothiocyanate-based labels. A pre-peak occurred with both examples mAbA3 and mAbB3, which were modified with Tm-*p*-SCN-Bz-DOTA. This phenomenon of the additional pre-peak in the FcRn affinity chromatography of IgGs, labelled with isothiocyanate-tags, has already observed. As the DoL is gradually increased, the pre-peak also increases [29]. Interaction with the stationary phase can be excluded, since mAbB3 shows no retention in a negative control with a sepharose (without FcRn) column (Fig 6). Our conclusion is that conjugation with an SCN-functionalized label creates a new variant that has, depending on DoL, a lower affinity to FcRn. Furthermore, mAbA3 showed a similar retention time to the unlabelled wild-type mAb_A_, whereby the identical label in mAbB3 caused a significantly higher affinity to FcRn.

### Heparin affinity chromatography

As a polysulfated polysaccharide, heparin is a strongly (poly-) negatively charged glucosaminoglycan. Such polymers are commonly found on cell surfaces of vascular endothelial cells and immune cells, such as monocytes or macrophages [30]. IgGs that bind to heparin are exposed of the risk of unspecific pinocytosis and thus the degradation of the protein [31]. Heparin chromatography offers a second way to control the influence of labels on changes in protein properties by charge-based glycocalyx interaction on a heparin affinity column [32]. Starting with low-salt condition, heparin chromatography retains antibodies even with a low affinity for heparin. The elution is driven by a salt gradient and separates proteins according to difference in their ionic binding strength.

The corresponding retention times of the labelled antibodies are shown in Table 4. mAbB2, which has been labelled with Alexa Fluor 488-maleimide, has a large discrepancy. This is not surprising since previous controls (MS and SEC) showed that the antibody is no longer intact after the labelling process. The NSP-labelled mAb from the mAb_A_ series differs most clearly from the unlabelled antibody. The significant increase in heparin affinity for mAbB3 (Tm-*p*-SCN-Bz-DOTA) is surprising. When incorporating a negatively charged DOTA-label, it would be assumed that the affinity for negatively charged heparin decreases, as can be observed for mAbA3 with the same label from the mAb_A_ series.

### FcRn/heparin correlation

**Fig 7:**
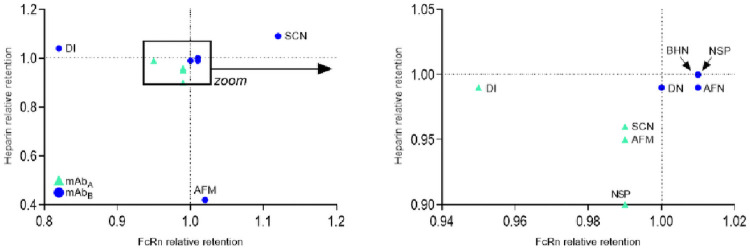
Correlation of FcRn and heparin column retention of labelled antibodies. Green triangle: mAb_A_ series, blue dot: mAb_B_ series. Left: full graphic, right represents the framed area. Dotted lines indicate a relative retention of 1.00. Label Code: NSP: *N*-succinimidyl propionate; AFM: Alexa Fluor 488-maleimide; SCN: Tm-*p*-SCN-Bz-DOTA; DI: direct iodination; AFN: Alexa Fluor 488-NHS; BHN: Bolton-Hunter-NHS; DN: DOTA-NHS.

A combination of FcRn and Heparin affinity chromatography strengthen an assessment of the quality of therapeutic antibodies after modification. The retention times of labelled and unlabelled protein in both affinity chromatographies, FcRn and heparin, are set in proportion according to S4 Formula to calculate a relative retention time of each labelled IgG (Table 5). A correlation of FcRn and heparin retention (Fig 7) identifies deviations in affinities for the unlabelled antibody. For the labelled IgG, a relative retention time of < 1 in FcRn affinity chromatography indicates a lower IgG:FcRn binding. In contrast to heparin affinity, a relative retention time of > 1 may be indicative of a higher unspecific uptake in cells.

**Table 5:** Calculated relative retention times.

*mAb #*	*Label Code*	*Relative t_R_ FcRn*	*Relative t_R_ Heparin*
mAb_A_	---	1.00	1.00
A1	NSP	0.99	0.90
A2	AFM	0.99	0.95
A3	SCN	0.99	0.96
A4	DI	0.95	0.99
mAb_b_	---	1.00	1.00
B1	NSP	1.01	1.00
B2	AFM	1.02	0.42
B3	SCN	1.12	1.09
B4	DI	0.82	1.04
B5	AFN	1.01	0.99
B6	BHN	1.01	1.00
B7	DN	1.00	0.99

Calculated relative retention times from FcRn and heparin affinity chromatography by the use of S4 Formula. Label Code: NSP: *N*-succinimidyl propionate; AFM: Alexa Fluor 488-maleimide; SCN: Tm-*p*-SCN-Bz-DOTA; DI: direct iodination; AFN: Alexa Fluor 488-NHS; BHN: Bolton-Hunter-NHS; DN: DOTA-NHS.

Direct iodinations (mAbA4 and mAbB4) lead to a significant decrease in FcRn affinity due to oxidation of the protein in the neonatal Fc receptor binding interface. All antibodies from the mAb_B_ series labelled with the *N*-hydroxysuccinimide technique (NSP, AFN, BHN, DN) are closely related in their correlation. In contrast, the only antibody from the mAb_A_ series that was labelled with the NHS technique (NSP) shows a deviation, although mAbA1 has a lower DoL (1.9) compared to mAbB1 (2.5).

## Conclusions

The main purpose of this investigation was to develop an *in vitro* assessment workflow to predict a change of physiological properties of antibodies after incorporation of a label. These combinations of several analytical methods reveal changes after label conjugation that might otherwise have remained undetected. Besides classical size-exclusion chromatography, the core element of this analysis package consists of correlating relative retention times (t _*R*_) for FcRn and heparin affinity chromatography of labelled mAbs with its congeners. So far, it has been a challenge to decide which shift in retention time is crucial for the application of the labelled protein in biological experiments. In a t _*R*_ range of 0.94 to 1.02 for FcRn and 0.90 to 1.05 for heparin affinity chromatography, however, no critical effect of the label is expected. These ranges have been tentatively selected and may shift with increasing empirical data collection.

It has been demonstrated that direct iodination can damage mAbs, which has been shown using FcRn affinity chromatography. During the labelling process, the protein is oxidized and leads to a lower affinity for the neonatal Fc receptor and consequently to a higher antibody clearance. An alternative to direct iodination is indirect iodination using the Bolton-Hunter reagent. The quality assessment after protein modification by conjugation of the iodinated Bolton-Hunter reagent shows a less pronounced change in the protein properties compared to direct iodination. Protein modifications with maleimide-based labels, which requires a previous partial reduction of the protein, can lead to an impairment of the quaternary protein structure. SEC analysis under denatured sample preparation conditions was indicative for free heavy and light chain after maleimide-based labelling. During the reduction step, cystine disulfide bonds are reduced to allow free Cys to conjugate with the labelling reagent. However, unconjugated cysteines are not re-oxidized and the antibody’s quaternary structure is only maintained through intermolecular forces, e.g., ionic and hydrogen bonds. At denaturing conditions caused by low pH and organic solvent, these bonds are released and, as there are no longer any covalent disulfide bridges, the antibody degrades into heavy and light chains. This could be of importance in an *in vivo* setting when, during FcRn recycling, the endosomal pH is dropping and the antibody is degraded instead of being reintroduced into circulation.

In conclusion, both type and degree of label of modified IgGs lead to changes in the physico-chemical protein properties. In addition, harsh labelling conditions can also impact the behaviour of labelled mAbs. Finally, identical labels on separate constructs of IgG_1_ can lead to different affinities for FcRn and heparin. This finding underlines the importance to assess the impact of the label for each new antibody construct individually and, unfortunately, the findings from this study cannot be taken as a general guide to which labelling strategy is best suited in all cases. Considering the assessment data, only 6 of the 11 modified mAbs in this study can be recommended for use in *in vitro* or *in vivo* experiments.

The benefits of conducting a quality assessment prior to mechanistic studies are manifold. Obviously, time and resources can be saved by minimising the risk of using unsuitable or even instable test molecules that generate questionable data. Another crucial advantage is the reduction of animal experiments by avoiding proteins that have been impacted by the labelling process. From an ethical point of view, this analytical workflow is a contribution to the 3Rs-principles, so that animal experiments that could lead to incorrect results or conclusions (with respect to human data/clinical trials) are not conducted.

This *in vitro* quality assessment has become the analytical standard for labelled antibodies at Roche. Therefore, this analysis package is an important tool for characterization of labelled antibodies that ensures reliable extrapolation to the respective unlabelled antibodies.

## Supporting information

S1 Graphical abstract (DOCX)

S1 Sequences Protein sequences of wild-type mAb_A_ and mAb_B_ containing LALA PG mutation. (PDF)

S1 Table Labelling distribution on light and heavy chain calculated by ion peak integration in mass spectrometry. Label distribution calculation of intact protein follows the probability of Poisson distribution (S1 Formula).
(PDF)

S2 Table Absorbance of fluorescent labelled antibodies. mAbs A2, B2, and B5 absorbances at 280 nm (A_280_) and 494 nm (A_494_). ε: molar extinction factor; d: path length of cuvette; c: protein concentration in molarity [M] and mass per volume [mg/mL]; DoL: degree of label.
(PDF)

S1 Formula Probability (P) of events for a Poisson distribution. λ is the average number of events per interval; e represents the Euler’s number (2.7182…); k takes values 0, 1, 2, 3, 4, …; k! is the factorial of k.
(PDF)

S2 Formula Calculation of molar protein concentration labelled with Alexa Fluor 488. Absorbance of the protein solution at 280 nm (*A*_280_) and 494 nm (*A*_494_) in a cuvette with *d* = 0.1 cm path length, and the corresponding molar extinction coefficient ε (*M*).
(PDF)

S3 Formula Calculation of degree of labelling (DoL) for fluorescence labelled conjugates based on spectrometric analysis. Absorbance of the protein solution at 494 nm (A_494_) in a cuvette with d = 0.1 cm path length, approximate molar extinction coefficient of Alexa Fluor 488 dye ε (AF488) = 71,000 cm^-1^ M^-1^ at 494 nm, molar protein concentration c (M) was calculated by the use of S2 Formula.
(PDF)

S4 Formula Calculation of relative retention time for FcRn and heparin affinity chromatography. (PDF)

S1 Fig Chromatograms of mAbA1 to mAbA4 from the mAb_A_ series. Left column: SEC; center column: FcRn affinity chromatography; right column: heparin affinity chromatography. X-axis: time in min; left Y-axis: absorbance at 280 nm in black; right Y-axis: absorbance at 494 nm in green.
(PDF)

S2 Fig Chromatograms of mAbB1 to mAbB4 from the mAb_B_ series. Left column: SEC; center column: FcRn affinity chromatography; right column: heparin affinity chromatography. X-axis: time in min; left Y-axis: absorbance at 280 nm in black; right Y-axis: absorbance at 494 nm in green.
(PDF)

S3 Fig Chromatograms of mAbB5 to mAbB7 from the mAb_B_ series. Left column: SEC; center column: FcRn affinity chromatography; right column: heparin affinity chromatography. X-axis: time in min; left Y-axis: absorbance at 280 nm in black; right Y-axis: absorbance at 494 nm in green.
(PDF)
