# Supplementary figures and images for: Functional in vitro assessment of modified antibodies: Impact of label on protein properties
Source: PLoS One. 2021 Sep 16;16(9):e0257342. doi: 10.1371/journal.pone.0257342 (PMC8445452; doi:10.1371/journal.pone.0257342)

**S1 Graphical abstract**

**
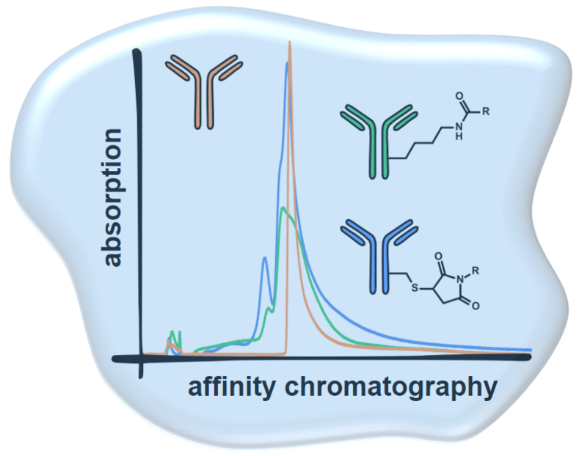
**

Supplement: S1 Graphical abstract — (DOCX) [file pone.0257342.s001.docx]
