# Supplementary material for: Functional in vitro assessment of modified antibodies: Impact of label on protein properties
Source: PLoS One. 2021 Sep 16;16(9):e0257342. doi: 10.1371/journal.pone.0257342 (PMC8445452; doi:10.1371/journal.pone.0257342)
Supplement: S1 Sequences — (PDF) [file pone.0257342.s002.pdf]

mAb$_A$ (wild type):

DIQMTQSPSS LSASVGDRVT ITCRASQDIK NYLNWYQQKP GKAPKLLIYY
SSTLLSGVPS RFSGSGSGTD FTLTISSLQP EDFATYYCQQ SITLPPTFGG
GTKVEIKRTV AAPSVFIFPP SDEQLKSGTA SVVCLLNNFY PREAKVQWKV
DNALQSGNSQ ESVTEQDSKD STYSLSSTLT LSKADYEKHK VYACEVTHQG
LSSPVTKSFN RGEC
QVQLVESGGG LVKPGGSLRL SCAASGFTFS DYAMSWIRQA PGKGLEWVSS
INIGATYIYY ADSVKGRFTI SRDNAKNSLY LQMNSLRAED TAVYYCARPG
SPYEYDKAYY SMAYWGQGTT VTVSSASTKG PSVFPLAPSS KSTSGGTAAL
GCLVKDYFPE PVTVSWNSGA LTSGVHTFPA VLQSSGLYSL SSVVTVPSSS
LGTQTYICNV NHKPSNTKVD KKVEPKSCDK THTCPPCPAP ELLGGPSVFL
FPPKPKDTLM ISRTPEVTCV VVDVSHEDPE VKFNWYVDGV EVHNAKTKPR
EEQYNSTYRV VSVLTVLHQD WLNGKEYKCK VSNKALPAPI EKTISKAKGQ
PREPQVYTLP PSRDELTKNQ VSLTCLVKGF YPSDIAVEWE SNGQPENNYK
TTPPVLDSDG SFFLYSKLTV DKSRWQQGNV FSCSVMHEAL HNHYTQKSLS
LSPGK

mAb$_B$ (LALA PG mutation):

EIVLTQSPGT LSLSPGERAT LSCRASQSVS SSYLAWYQQK PGQAPRLLIY
GASSRATGIP DRFSGSGSGT DFTLTISRLE PEDFAVYYCQ QYGSSPLTFG
QGTKVEIKRT VAAPSVFIFP PSDEQLKSGT ASVVCLLNNF YPREAKVQWK
VDNALQSGNS QESVTEQDSK DSTYSLSSTL TLSKADYEKH KVYACEVTHQ
GLSSPVTKSF NRGEC
EVQLLESGGG LVQPGGSLRL SCAASGFTFS SYAMSWVRQA PGKGLEWVSA
ISGSGGSTYY ADSVKGRFTI SRDNSKNTLY LQMNSLRAED TAVYYCAKGS
GFDYWGQGTL VTVSSASTKG PSVFPLAPSS KSTSGGTAAL GCLVKDYFPE
PVTVSWNSGA LTSGVHTFPA VLQSSGLYSL SSVVTVPSSS LGTQTYICNV
NHKPSNTKVD KKVEPKSCDK THTCPPCPAP EAAGGPSVFL FPPKPKDTLM
ISRTPEVTCV VVDVSHEDPE VKFNWYVDGV EVHNAKTKPR EEQYNSTYRV
VSVLTVLHQD WLNGKEYKCK VSNKALGAPI EKTISKAKGQ PREPQVYTLP
PSRDELTKNQ VSLTCLVKGF YPSDIAVEWE SNGQPENNYK TTPPVLDSDG
SFFLYSKLTV DKSRWQQGNV FSCSVMHEAL HNHYTQKSLS LSPGK

S1 Sequences: Protein sequences of wild-type mAb$_A$ and mAb$_B$ containing LALA PG mutation.
