# Supplementary material for: Functional in vitro assessment of modified antibodies: Impact of label on protein properties
Source: PLoS One. 2021 Sep 16;16(9):e0257342. doi: 10.1371/journal.pone.0257342 (PMC8445452; doi:10.1371/journal.pone.0257342)
Supplement: S1 Table — Label distribution calculation of intact protein follows the probability of Poisson distribution (S1 Formula). (PDF) [file pone.0257342.s003.pdf]

mAb	Label light chain [%]				Label heavy chain [%]				Label intact protein [%]				
#	0	1	2	3	0	1	2	3	0	1	2	3	4
A1	54	38	8	0	66	29	5	0	16	29	27	17	8
A2	43	57	0	0	25	64	10	1	6	16	23	22	16
A3	66	32	2	0	80	20	0	0	33	37	21	8	2
A4	59	38	3	0	65	30	5	0	19	31	26	15	6
B1	57	34	9	0	48	30	11	7	8	21	26	21	13
B2	58	42	0	0	54	44	2	0	17	30	27	16	7
B3	66	31	3	0	49	41	10	1	13	27	27	18	9
B4	77	20	3	0	24	39	26	12	5	14	22	22	17
B5	71	26	2	0	69	26	5	0	27	35	23	10	3
B6	65	34	1	0	63	33	4	0	21	33	25	13	5
B7	91	9	0	0	66	31	3	0	40	37	17	5	1

S1 Table: **Labelling distribution on light and heavy chain calculated by ion peak integration in mass spectrometry.** Label distribution calculation of intact protein follows the probability of Poisson distribution (**S1 Formula**).
