# Supplementary material for: Functional in vitro assessment of modified antibodies: Impact of label on protein properties
Source: PLoS One. 2021 Sep 16;16(9):e0257342. doi: 10.1371/journal.pone.0257342 (PMC8445452; doi:10.1371/journal.pone.0257342)
Supplement: S2 Table — mAbs A2, B2, and B5 absorbances at 280 nm (A280) and 494 nm (A494). ε: molar extinction factor; d: path length of cuvette; c: protein concentration in molarity [M] and mass per volume [mg/mL]; DoL: degree of label. (PDF) [file pone.0257342.s004.pdf]

mAb #	A_{280}	A_{494}	ε [cm^{-1} M^{-1}]	d [cm]	c [M] ; [mg/mL]	DoL
A2	0.185	0.416	198,420	0.1	6.5×10^{-6}; 0.95	9.0
B2	0.188	0.296	213,890	0.1	7.8×10^{-6}; 1.11	5.3
B5	0.210	0.125	213,890	0.1	9.9×10^{-6}; 1.41	1.7

S2 Table: **Absorbance of fluorescent labelled antibodies**. mAbs A2, B2, and B5 absorbances at 280 nm (A_{280}) and 494 nm (A_{494}). ε: molar extinction factor; d: path length of cuvette; c: protein concentration in molarity [M] and mass per volume [mg/mL]; DoL: degree of label.
