# Supplementary material for: Functional in vitro assessment of modified antibodies: Impact of label on protein properties
Source: PLoS One. 2021 Sep 16;16(9):e0257342. doi: 10.1371/journal.pone.0257342 (PMC8445452; doi:10.1371/journal.pone.0257342)
Supplement: S1 Formula — λ is the average number of events per interval; e represents the Euler’s number (2.7182…); k takes values 0, 1, 2, 3, 4, …; k! is the factorial of k. (PDF) [file pone.0257342.s005.pdf]

$$P(k) = \frac{\lambda^k * e^{-\lambda}}{k!}$$

S1 Formula: **Probability (*P*) of events for a Poisson distribution**. λ is the average number of events per interval; *e* represents the Euler's number (2.7182…); *k* takes values 0, 1, 2, 3, 4, …; *k*! is the factorial of *k*.
