# Supplementary material for: Functional in vitro assessment of modified antibodies: Impact of label on protein properties
Source: PLoS One. 2021 Sep 16;16(9):e0257342. doi: 10.1371/journal.pone.0257342 (PMC8445452; doi:10.1371/journal.pone.0257342)
Supplement: S2 Formula — Absorbance of the protein solution at 280 nm (A280) and 494 nm (A494) in a cuvette with d = 0.1 cm path length, and the corresponding molar extinction coefficient ε (M). (PDF) [file pone.0257342.s006.pdf]

$$c(M)_{\mathrm{AF-mAbs}} = \frac{[A_{280} - (A_{494} \ * \ 0.11)]}{\varepsilon(M) \ * \ d}$$

S2 Formula: **Calculation of molar protein concentration labelled with Alexa Fluor 488.** Absorbance of the protein solution at 280 nm (A_{280}) and 494 nm (A_{494}) in a cuvette with d = 0.1 cm path length, and the corresponding molar extinction coefficient ε (M).
