# Supplementary material for: Functional in vitro assessment of modified antibodies: Impact of label on protein properties
Source: PLoS One. 2021 Sep 16;16(9):e0257342. doi: 10.1371/journal.pone.0257342 (PMC8445452; doi:10.1371/journal.pone.0257342)
Supplement: S3 Formula — Absorbance of the protein solution at 494 nm (A494) in a cuvette with d = 0.1 cm path length, approximate molar extinction coefficient of Alexa Fluor 488 dye ε (AF488) = 71,000 cm-1 M-1 at 494 nm, molar protein concentration c (M) was calculated by the use of S2 Formula. (PDF) [file pone.0257342.s007.pdf]

$$DoL_{\text{AF}-\text{mAbs}} = \frac{A_{494}}{\varepsilon\,(\text{AF488}) * c\,(M) * d}$$

S3 Formula: **Calculation of degree of labelling (DoL) for fluorescence labelled conjugates based on spectrometric analysis.** Absorbance of the protein solution at 494 nm (A_{494}) in a cuvette with d = 0.1 cm path length, approximate molar extinction coefficient of Alexa Fluor 488 dye ε (AF488) = 71,000 cm^{-1} M^{-1} at 494 nm, molar protein concentration c (M) was calculated by the use of **S2 Formula**.
