# Supplementary material for: Functional in vitro assessment of modified antibodies: Impact of label on protein properties
Source: PLoS One. 2021 Sep 16;16(9):e0257342. doi: 10.1371/journal.pone.0257342 (PMC8445452; doi:10.1371/journal.pone.0257342)
Supplement: S4 Formula — (PDF) [file pone.0257342.s008.pdf]

$$t_{relative\ labelled} = \frac{t_{labelled}}{t_{unlabelled}}$$

S4 Formula: **Calculation of relative retention time for FcRn and heparin affinity chromatography.**
