# Supplementary material for: Functional in vitro assessment of modified antibodies: Impact of label on protein properties
Source: PLoS One. 2021 Sep 16;16(9):e0257342. doi: 10.1371/journal.pone.0257342 (PMC8445452; doi:10.1371/journal.pone.0257342)
Supplement: S1 Fig — Left column: SEC; center column: FcRn affinity chromatography; right column: heparin affinity chromatography. X-axis: time in min; left Y-axis: absorbance at 280 nm in black; right Y-axis: absorbance at 494 nm in green. (PDF) [file pone.0257342.s009.pdf]

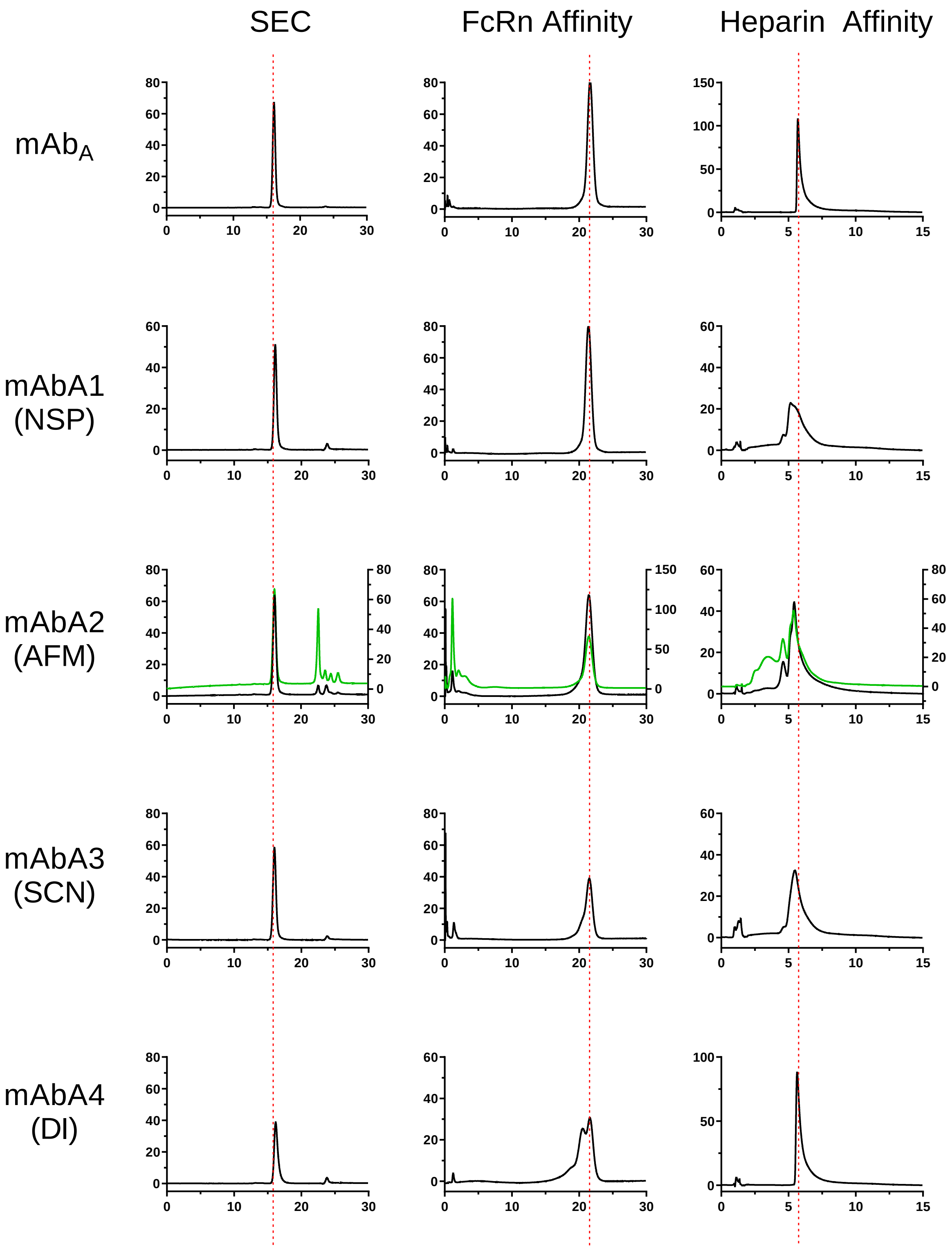

S1 Fig: **Chromatograms of mAbA1 to mAbA4 from the mAbA series.** Left column: SEC; center column: FcRn affinity chromatography; right column: heparin affinity chromatography. X-axis: time in min; left Y-axis: absorbance at 280 nm in black; right Y-axis: absorbance at 494 nm in green.
